# “It’s all about the money”: an interpretive description of embedding physical therapy-led falls prevention group exercise in long-term care

**DOI:** 10.1186/s12877-022-03722-z

**Published:** 2023-01-11

**Authors:** Elizabeth Binns, Felicity Bright, John Parsons, Kathy Peri, Lynne Taylor, Ngaire Kerse, Denise Taylor

**Affiliations:** 1 Physiotherapy Department, Auckland University of Technology, Wellesley Campus, Private Bag 92006, 1142 Auckland, New Zealand; 2 Health and Rehabilitation Research Institute, Auckland University of Technology, Auckland, New Zealand; 3 Centre for Person Centred Research, Auckland University of Technology, Auckland, New Zealand; 4 School of Nursing, University of Auckland, Auckland, New Zealand; 5 Department of Exercise Sciences, University of Auckland, Auckland, New Zealand; 6 School of Population Health, University of Auckland, Auckland, New Zealand; 7 New Zealand Dizziness & Balance Centre, Auckland, New Zealand

**Keywords:** Falls prevention, Older adults, Long-term care, Qualitative research

## Abstract

**Background:**

Falls prevention interventions are effective for community dwelling older adults however, the same cannot be said for older adults living in long-term care (LTC). The Staying UpRight (SUp) randomized controlled trial was designed to test the effectiveness of a progressive strength and balance group exercise program delivered to LTC residents. This paper explores the factors impacting LTC providers’ decisions to continue the program on completion of the funded trial period.

**Methods:**

A qualitative study using an Interpretive Description approach. Semi-structured interviews and focus groups were conducted with 15 LTC staff involved in the randomized controlled trial. Data were analysed using conventional content analysis.

**Results:**

Practice change occurred following participation in the trial with some facilities starting exercise groups, some increasing the number of exercise groups offered and physical therapists selecting elements of the program to adopt into their practice. Decisions about continuing with SUp as designed were constrained by organizational decisions regarding funding and resources. Three factors were identified which informed decision-making: business models and philosophies, requirements for evidence, and valuing physical therapy.

**Conclusions:**

Managers and facilitators adapted SUp by selecting and delivering components of the program in response to the changes they had observed in participating residents. However, our findings highlight that while SUp was valued, the tight financial environment created by the current funding model in New Zealand did not support funding physical therapist delivered falls prevention exercise programs in LTC. This study may provide policy makers with important information on changes needed to support falls prevention service delivery in LTC.

**Trial registration:**

This study is a sub-study of a randomized controlled trial which was registered to the Australian New Zealand Clinical Trials Registry ACTRN12618001827224 on 09/11/2018. Universal trial number U1111-1217-7148.

**Supplementary Information:**

The online version contains supplementary material available at 10.1186/s12877-022-03722-z.

## Background

The fall rate for older adults living in long-term care (LTC) is higher than community dwelling older adults [1, 2], which is a reflection of residents’ high levels of physical frailty and disability; both high risk factors for falls [3]. The LTC environment may compound physical impairment through staff limiting the physical activity of residents they consider to be fall-prone or considering ‘walking’ to be the domain of physical therapists, leading to increased sedentary behaviour and lower limb muscle weakness in residents [4, 5].

To be admitted into LTC, an older adult is assessed as no longer able to live independently at home [6]. The International Resident Assessment Instrument (interRAI) Home Care is a mandated component of this assessment and determines what level of care is needed [7]. A national contract (Age-Related Residential Care Services Agreement [8]) between the national health authority (New Zealand Ministry of Health) and LTC facilities defines the Government funded care services provided to a resident. Services covered by the agreement include an individualized care plan based on the interRAI LTC facilities assessment. The agreement specifies a facility must have a falls prevention policy and assess, prevent and manage falls however, it does not require that there is physical therapy input into falls prevention [9, 10].

The Staying UpRight (SUp) falls prevention exercise program was developed to address the fall risk factors of decreased lower limb strength and impaired balance in older adults living in LTC. The program was informed by clinical experience, previous falls prevention research, and an understanding of the physiological systems of balance and principles of rehabilitation (task specificity, progression and overload) [11–14]. The SUp exercise program (intervention) was delivered by physical therapists and the dose matched chair exercise program, Flex and Stretch (control), was delivered by LTC activities staff. A pilot study found improvements in physical function, no adverse events, and that the program was acceptable to participants and staff [15].

There is limited evidence regarding the sustainability of falls prevention programs in LTC. There is a lack of evidence from the experience of different levels of staff within the same LTC facility (senior management, onsite management, and frontline staff) examining the sustainability of falls prevention interventions. The field of implementation science currently focusses more on the initial uptake of evidence-based practice rather than whether it is sustained. This means we lack an understanding of what happens over time [16]. The limited evidence from community falls prevention programs suggests that health practitioners experience personal and interpersonal influences, clinical barriers and limitations of research evidence as barriers to implementation [17]. At an organizational level funding has been identified as a critical factor for sustainability [18].

This paper reports a qualitative study completed as part of an effectiveness-implementation hybrid type 1 study [19]. This study ran alongside the Staying UpRight (SUp) randomized controlled trial (RCT) which assessed the effectiveness of a 12-month strength and balance group exercise program compared with a control program (Flex and Stretch) [20]. The RCT findings will be published separately. Qualitative studies are the most common design used to evaluate sustainability of evidence-based interventions [21]. Our qualitative study explored what factors influenced the maintenance of SUp as usual practice. In this paper, we report the perspectives of managerial and clinical LTC staff.

## Methods

### Study design

This study utilises an Interpretive Description (ID) [22] methodology. This qualitative approach seeks to provide insight into practice-oriented issues and generate findings which could be applied in practice settings. The study is reported in accordance with the Standards for Reporting Qualitative Research [23]. Ethical approval was given by the New Zealand (NZ) Health and Disability Ethics Committee (HDEC) (18/NTB/151/AM04). This study is a sub-study of the RCT which was registered to the Australian New Zealand Clinical Trials Registry ACTRN12618001827224 on 09/11/2018. Universal trial number U1111-1217-7148.

### Sampling and recruitment

Three NZ LTC organizations involved in the RCT were purposively sampled, seeking variation across business structure: one publicly listed (‘for-profit’) company, one private company (‘for-profit’) and one charitable organization (charity). Grouping facilities by organization enabled exploration of whether organizational influence impacted SUp being embedded in facilities. The sample were staff from the three organizations. Once organization consent was gained, researchers sampled for maximum diversity in participants by job role: senior management, onsite management and clinical staff. Eligible staff were emailed an invitation to participate by researchers. All participants gave written informed consent. All methods were carried out in accordance with relevant guidelines and regulations.

### Data collection

Interviews and focus groups took place between April and August 2021 in person or via Zoom. Two experienced qualitative researchers (JP and KP) who were not involved in the trial conducted the interviews and focus groups. Interviews were conducted with senior management and onsite management staff. These allowed for detailed exploration of organizational contexts and discussion of commercially sensitive information. Focus groups were held with exercise group facilitators to allow exploration of a breadth of experiences and explore similarities and differences across practice settings. Separate focus groups were held specific to the exercise group (SUp and Flex and Stretch). One physical therapist had an individual interview as no other therapists attended the planned focus group. Three unplanned focus groups occurred when management and clinical staff at the same facility chose to be interviewed together. Questions followed an interview guide informed by Curran [19], supplemented with follow up questions and prompts to deeply explore participant experiences (Additional file 1). In response to low facilitator participation and COVID lockdowns, an amendment was approved by HDEC on 23 September 2021 (ref 2021 AM 7851), to use facilitator emails sent during the RCT that discussed the classes, as data.

### Data analysis

Interviews and focus groups were recorded and transcribed. Transcripts and high-level summaries were sent back to participants for confirmation of accuracy and meaning intent [24]. De-identified transcripts were organised and coded in NVivo, release 1.0 (QSR International, Melbourne, Australia) [25]. We used conventional content analysis and followed the steps of listening to the recordings, reading, re-reading, coding individual transcripts, and refining codes as data analysis progressed. These were defined within a codebook. We worked across multiple transcripts and inductively formed categories [26]. Emails were also organised and coded in NVivo. Conventional content analysis was used and followed the steps of reading and re-reading, coding with codes generated from the interview and focus groups analysis and further code development. Once coded, initial themes were generated by category handling, specifically, writing themes on paper and manually arranging them [27]. These were represented in a concept map for final discussion between research team members [28]. Direct quotes were used to illustrate points made to ensure confirmability. Maximum variation sampling enabled data triangulation of interviews with multiple participants and constant comparison was used to ensure not privileging one account over another.

To aid rigour, a reflexive approach was taken throughout the analysis process to acknowledge the researcher’s professional training, clinical experience, previous research, and role in developing the RCT exercise programs. The involvement of independent interviewers and co-authors with methodological expertise added rigour, as did processes such as negative case analysis, triangulation, journaling and constant reference to raw data.

## Results

Fifteen people took part (Table 1). Five interviews were held, lasting between 15 and 34 minutes and five focus groups, lasting between 19 and 41 minutes. Twenty-four facilitators (*n* = 19 SUp facilitators and *n* = 5 Stretch and Flex facilitators) gave retrospective consent for their emails to be included in the data analysis. Quotes are reported with a participant identifier; SM (senior management), Mgmt (onsite management) and FAC (exercise group facilitator).

**Table 1:** Characteristics of the interview and focus group participants (*n* = 15)

Characteristics	n (%)
Gender	
Male	1 (7)
Female	14 (93)
Role	
Senior Management	4 (27)
Onsite Management	5 (33)
Staying UpRight Facilitator (physical therapist)	4 (27)
Flex and Stretch Facilitator	2 (13)
Professional background	
Nurse	8 (53)
Physical Therapist	6 (40)
- facility Employee (NZ registered)	1
- facility Employee (overseas trained, not NZ registered)	2
- contractor (NZ registered)	3
Unknown	1 (7)
Years working in LTC	
< 5	3 (20)
5-10	6 (40)
10+	5 (33)
Unknown	1 (1)

The decision-making of management staff was key in embedding falls prevention in organizations. The decisions made regarding use of resource and funding influenced what types of falls prevention approaches were used and who they were delivered by. Decision-making regarding how individual programs were delivered happened at clinician level – the activities co-ordinator or physical therapist. Decision-making appeared to be informed by several factors: business models and philosophies, requirements for evidence, and valuing the contribution of physical therapy. Table 2 illustrates the main factors and subtopics that emerged from the interview, focus group and email data.

**Table 2:** Summary of findings

Themes	Sub-themes
Business models and philosophies	Driven for profit versus being driven by care No specified model of care
Requirements for evidence	Knowledge of results required for financial investment Anecdotal evidence informed practice change
Valuing physical therapy	The invisible skillset Time equals money and money equals time

### The influence of business models and philosophies on embedding SUp

An organization’s ability and, perhaps more significantly, willingness to embed SUp as a sustained usual care program appeared to be informed by their business model and philosophy.

#### Being driven by the need to generate a profit versus being driven to provide care

For-profit organizations needed to make a profit for shareholders and required their facilities to be “fiscally responsible” (SM#2). Government funding was considered insufficient, with one senior management participant saying: “the amount we get per day doesn’t look after the residents right now” (SM#1). Some facilities were not financially viable. Organizations used profits from the sale of independent units co-located in the retirement village (self-care apartments and villas) for facility operational costs however, doing so decreased organization profits. One senior management participant observed that “…unless the government recognises (the shortfall), there’s gonna be a lot of providers go out of business. Particularly those that don’t have villages. [to embed SUp in practice] We would have to actually think about how we funded it in the tight environment that we’re in at the moment”. In this financial context, physical therapist delivery of SUp was perceived as a cost. This brought tensions. Whilst valued for being “resident focused and quality of life focused” (SM#1), delivery of SUp was balanced against organizational finances, with participants saying “we have to be able to afford them [SUp classes] and not go down the gurgler” (SM#1) and “I would absolutely love to have something like this (SUp) in, but it’s the matter of the money” (SM#2). Another senior management participant discussed weighing up the increase of a resident’s wellbeing against their length of stay in a facility to determine the return on investment in SUp, “should it be an investment that we make to ensure that our rest home level care residents are more well? But then do we get them all well and they go home? (laughter) That’s one side of the coin, but the other side of it is, get them all well and more mobile so that their quality of life is better and they live longer in a lovely environment with us” (SM#3). This comment was immediately followed by an expression discomfort about basing a decision to improve a person’s quality of life on financial gain, “[it] sounds pretty horrible” (SM#3).

In contrast to the for-profit organizations, the charity LTC organization received government and charitable funding. The charity required only that the organization provide care for those in need. This saw the organization foreground the well-being of residents alongside prudent financial management. Business decision-making was guided by principles of “promoting well-being” (Mgmt#5) and feedback gathered through surveys and resident focus groups. This created an environment where management did not feel financially constrained and could approach the Board for new initiative funding. The Board’s view on the positive contribution made by physical therapy to resident’s wellbeing was well known. The management participant reflected on the possibility of continuing SUp as designed if it was found to be effective, “is there a really good argument now to actually increase physio not just hold status quo” (Mgmt#5).

The for-profit organizations drive for profitability meant management had an acute awareness of cost. This led to tight budgeting which constrained the uptake of any new initiatives in the absence of specific funding. The charity organization’s drive for resident well-being led to regular review of service delivery. Services were updated as part of business as usual to deliver better resident outcomes. The charity, in contrast to the for-profit organizations, had a mechanism for management to gain financial support for new initiatives if needed.

#### No model of care specified to determine the delivery of care

The NZ Government service specifications outline *what* is to be delivered in LTC but not *how* it should be delivered. Whilst a falls prevention policy was a requirement, the assessment and management of falls was determined by the LTC facility. This included to what extent physical therapy was involved.

Organizations were acutely aware of the NZ Government service specifications and what they were paid to provide. They also knew that the configuration and delivery of services were not dictated by the Government. A senior management participant quoted the service specification almost word for word, “there’s a responsibility to ensure that older people remain active and have access to doing active things…there’s not a specific model of care, if you like. And so the variation in the sector, not just for [organization name] but for everyone, is significant” (SM#1). Management participants knew that physical therapy was not in the service specifications. Most facilities had access to physical therapy however, this was variable within and across organizations, “physio is a bit of a challenge in the aged care sector and for us particularly it’s quite variable. So it is something that I think has more, has value, but there isn’t a really clear program of how physios would actually interface with aged care” (SM#1).

Usual therapy for residents was planned and supervised by physical therapists but often delivered by unregistered health professional staff: “[We] do a thorough physio assessment on them and then at facilities where we have assistants, we hand on to them mostly, for the ongoing care” (FAC#3). A management participant echoed “[physical therapist name] is more of like overseeing what’s happening in the care home. So that’s why she has a physio assistant. So the physio assistant can continue the plan. She’s like the brain and the physio assistant is the skills” (Mgmt#3). Several participants expressed the personal conflict experienced by knowing what residents needed wasn’t necessarily provided, “it comes back to my core values and belief that we don’t have a reablement pathway in aged residential care at the moment. And it’s not even everybody that needs it, is it? But for those that do, I think it’s unfair that they don’t really get that” (SM#3). Similarly, another said: “they [residents] often come in very deconditioned and we build them up. So being able to actually do some really good physio intervention as part of that gets them into a much better health state. So it should be part of what we do, frankly” (SM#2).

Senior and management participants’ desire to care for people revealed the ethos of their clinical background. They understood how the service specifications impacted on service delivery through what was and wasn’t specifically funded and how this effected outcomes for residents in their care.

### Requirements for evidence

All participants considered SUp was valuable, but they wanted evidence that falls were prevented in order to support a case for SUp being integrated into everyday practice. This was needed for organizational resourcing, and to support individual therapists to change their practice.

#### Knowledge of results required for financial investment

For organizations to support routine SUp provision, participants in management roles needed to “see what the results are first” (SM#2). They stated that having data would strengthen a business case for ongoing funding. A manager said, “it would be tremendous to see the results of the overall research because it always just reinforces that you’re on the right track with something. And it should drive business decisions,” (Mgmt#5) and knowing that “(SUp) would be evidence based and proven” (SM#4) would be crucial to being able to fund the delivery of SUp after the program of research finished. There was also an awareness of the economics of being able to deliver the program in a group setting: “I think group exercise session is probably a much better bang for your buck, because you’re covering off a large group” (SM#2). This perhaps reflects the dominant discourse of evidence-based practice entrenched in health care services.

#### Anecdotal evidence informed practice change

Clinicians, predominantly, considered the practice-based evidence they observed through participating in the RCT was sufficient to support using SUp in usual care. In particular, they drew on observations of individual residents, referring to changes in (1) residents’ capabilities: “one of the ladies walked all the way up from the downstairs wing and walked back - previously she was using a wheelchair to get to and from the class” (FAC#4); (2) increased fitness and balance: “I was very pleasantly surprised how rapidly I could get them up to 60 minutes of exercise and how far I could get in the difficulty of exercises challenging their balance” (FAC#8); and (3) resident engagement: “residents had asked for more exercises” (FAC#6). Some physical therapy participants built on this, adding classes to usual care where previously none were available or increasing the number of classes offered and applying concepts from SUp within these: “It’s hard to teach an old dog new tricks, but we have learnt some… it has made us realise that we can push them” (FAC#2). The engagement of the physical therapists themselves was seen as positive and convinced some management participants to support practice change, with one reflecting: “physios don’t change stuff without good evidence” (Mgmt#5), while another requested the physical therapist run more classes. While physical therapy SUp facilitators could see changes in individual residents’ functional abilities, management participants needed to measure the change across all those who took part in the SUp program and were specifically focused on the falls prevention outcome. Observing changes in participants and perceiving the program produced better outcomes for residents resulted in clinicians being more open to change. However, this resulted in clinicians incorporating concepts of SUp into usual care rather than management looking for additional funding to continue the SUp program.

### Valuing the contributions of physical therapy in falls prevention

Physical therapists were part of the multidisciplinary team in all facilities, but their skillset was utilised differently between the for-profit and charity organizations. In the for-profit organizations their input in falls prevention was minimal.

#### The invisible skillset of physical therapy in LTC

Physical therapy input was viewed positively at the charity, reflected by the manager observing “it’s such a drawcard for lots of families, having such a proactive physio team” (Mgmt#5). However, in the for-profit organizations physical therapists were predominantly “limited to assessment and advice” (FAC#4), contributing to multidisciplinary resident assessments and care plans. A senior management participant observed that “the physios do more paperwork than they do time with the residents doing walking programs” (SM#4). This perhaps meant managers and other staff did not experience and understand what falls prevention skills physical therapists had. Instead, falls prevention practices were a set of discrete tasks completed by a nurse or caregiver, mainly focused on the physical environment. If residents were having recurrent falls, physical therapists were sought for advice on how to manage the environment rather than for therapeutic intervention, as one manager illustrated: “when the resident had a fall, usually the clinical team will send an email to [physio name], “Can you please review this transfer plan? Is there something else that we can do?”” (Mgmt#3). This then contributed to senior management participants’ expectations not being met and they questioned the value of physical therapy. One commented: “Whenever I … do any clinical reviews of [residents], their physio assessments and the physio input into their plan is nothing like what I think it could be or should be. So it comes down to telling them, ‘Yes, carry on with the walker’. Well the nurse could’ve figured that out. So I don’t see a great depth of investment. Or individual planning.” (SM#3). The clinical reasoning of physical therapists appeared to be invisible to managers but was evident when physical therapists described how demanding it was to challenge a participant “to their level”. Observations and ongoing clinical decisions needed to be made during each SUp class, “you need to make a quick assessment of who is safe to stand up and try standing on one leg” (FAC#4). This demonstrated how assessment and clinical reasoning skills are central to individualising SUp and supporting individual progress. However, if this skillset is not usually recognised within an organization, this may not be ‘seen’ by those who make the financial decisions.

#### Time equals money and money equals time

All physical therapists in the for-profit organizations were contracted on an hourly basis. Any increase in physical therapist time had budgetary impact. The SUp research funded the physical therapists’ time and enabled physical therapists to prioritise SUp classes in their workload. In everyday practice, often group classes were cancelled or given to an assistant to take: “an exercise class is often the first thing to go off my list if I have lots of new referrals or someone very acute” (FAC#4). One physical therapist reflected on the possibility of continuing to provide SUp, saying “there’s no way they would be likely to allow me an hour out of my contracted hours… I couldn’t afford that much time. I would just get way too far behind on all my other stuff” (FAC#3). However, in the charity, physical therapy was available to all with the onsite physical therapy gym open to treat residents in the morning. In the afternoon physical therapists visited residents who “needed to be seen individually” (FAC#2). There appeared to be no cost sensitivity in the charity. The value the Board placed on physical therapy was reflected in physical therapists being employed on staff and well resourced. As such, the cost of physical therapy was already incurred “…we just know we fund physio…our team have just absorbed it” (Mgmt#5); this perhaps made it easier to make changes to physical therapist programs and for SUp to be maintained after the research program finished.

To deliver SUp without additional funding in the for-profit organizations, management participants looked for workarounds that would mean the program was not delivered by physical therapists as designed and tested in the RCT. Even when value was seen, the cost was considered: “…I think there’s absolute value in it, it’s just about how we do it, what the workforce’s availability is and what the cost is to the sector” (SM#1). To this end, the management preference was to deliver SUp using diversional therapists or activity coordinators already employed so they wouldn’t have to pay for a physical therapist. When a physical therapist SUp facilitator asked a manager about SUp continuing: “…he [the manager] said yes to carrying on [SUp] but he thought that his activity coordinators were able to take over both sessions a week so he wouldn’t have to pay for a physio” (FAC#7). Another appeared resigned, saying “It is a bit of a pity but what I expected as they don’t like spending money on physio” (FAC#5). This led to not wanting to deliver the program with FAC#5 saying, “I don’t imagine [name] will pay for me to continue sadly as the classes literally double the hours I get there!”. Contracted physical therapists were acutely aware of their time costs and the impacts of funding. The contrast of contracting versus employing physical therapists to work in facilities demonstrated spending as little as possible to meet the requirements of the service agreement and create a profit versus not being profit driven. This highlights how business models and philosophies shaped care decision-making.

## Discussion

The study results revealed that organizational budgets, and underpinning contractual and financial requirements, influenced whether managers and/or physical therapists considered it was possible to embed a falls prevention exercise program as standard practice in LTC after the cessation of the research program. Identifying these factors highlights to designers of future falls prevention initiatives that on-going funding, as an aspect of delivery and maintenance, must be considered. This evidence also illustrates to policy makers that service specifications are used as drivers of care delivery and that identifying and addressing key health issues such as falls prevention should be considered in the wording of contractual documents.

Healthcare can be considered a complex adaptive system, as it comprises different components that are dynamically inter-related, changing in response to events. In this study facilities, organizations and the Government are system components however, each is also its own system. With this in mind, a complex adaptive systems view was taken and SUp considered as an event occurring in the complex adaptive system of the NZ health system [29]. While the study findings are contextual to NZ due to the funding model, the findings may be translatable to other countries when considering service delivery of falls prevention programs in LTC.

In NZ, means testing is used to determine the amount of government subsidy paid to a LTC facility for a person’s residency. Most residents receive a form of subsidy [30]. Government funding varies for residents assessed as requiring low dependency, high dependency and dementia level care, but is not based on the individual care needs of the resident as assessed by the interRAI. All study participants with budget responsibility described not only the cost of physical therapy as a barrier but also the larger issue of insufficient government funding for the increasing level of care residents needed. Their experiences echo an industry report that sought to update the funding model for LTC from the current three broad levels of low dependency, high dependency and dementia level care [31]. This report proposed a case-mix funding model and validated the use of interRAI Resource Utilisation Group (RUG-III) data to better reflect the need and funding required to care for each resident [31]. Following publication of the report, a NZ Government commissioned review of the LTC funding model recommended the use of interRAI RUG-III as a more sensitive model for allocating funding [32]. With each resident assessed with the interRAI 6 monthly, adopting a case-mix funding model would match the resident’s current care needs on an ongoing basis [33]. The review recommendations have not yet been adopted. The rationalisation of health resources in the face of an ageing population will be an ongoing pressure as LTC is increasingly used for end of/late life care, with residents living in LTC for an average of 18 months but the mortality rate within 1 month of admission reported as high as 36.5% in NZ [32, 34]. Providing the level of care needed at this stage of life is resource intensive. The senior management participants’ call for more funding to embed SUp rather than redistributing resources to falls prevention is understandable.

Considering the financial environment, growing care demands from increasingly frail residents and the drive for fiscal responsibility, if physical therapy is not understood and valued, it is likely that management either will seek cheaper methods of delivering SUp or not continue it at all. Delivering SUp without increasing costs saw participants adapting the intervention for their context [35]. Physical therapist participants adapted SUp content by selecting elements of the program and integrating concepts in their current workload, while management participants’ adapted SUp delivery by using unregistered (cheaper) healthcare professionals. Using unregistered healthcare professionals to deliver falls prevention exercise is not uncommon in the community and can be effective [36, 37]. However, in previous LTC research, unregistered healthcare professionals were trained to deliver a manualised falls prevention exercise program and exercised participants in sitting “for safety”, removing the element of standing balance, a critical component of falls prevention exercise [38, 39]. This suggests that more physical therapist input may be required to train and maintain program delivery by unregistered healthcare professionals, negating cost savings by not using physical therapists to deliver programs. The lack of parameters for physical therapy service in LTC creates the potential for providers to deliver to the minimal contractual obligation. A NZ survey of 373 facilities reported only 16 physical therapists were employed but 55 assistant physical therapists and 895 activities co-ordinators [40]. However, the annual NZ physical therapy workforce survey reported 111 physical therapists working in LTC [41]. Rather than employ physical therapists like the charity in this study, it appears that most facilities contract physical therapists and employ activities co-ordinators to carry out physical therapy plans and provide “activity” to meet government service specifications. This may also reflect the shift from socially oriented and charitable providers to large corporations now providing the majority of LTC beds and needing to generate investor profit [42]. A lack of parameters to guide delivery of physical therapy services in LTC is not unique to NZ and has been found to vary widely between countries [43]. The Netherlands utilised physical therapy the most; with a focus on rehabilitation and the goal of discharging people back to their own homes from LTC. In Canada, UK, Denmark, Italy and Japan some LTC facilities had no physical therapy services. Government funding appears to be the common denominator for determining physical therapy utilisation with the Netherlands Government fully funding physical therapy and the UK, Canadian and NZ governments only partially funding physical therapy in LTC [43, 44].

The research funding allowed the delivery SUp to be prioritised; that the program ceased when funding ended was not surprising. When an intervention (SUp) is introduced to a complex system (LTC), the system realigns to accommodate the new event, often at the expense of another component within the system. In the LTC facilities that ran exercise programs prior to SUp, they were typically only 30 minutes long. For the SUp classes to be accommodated, these class times were gradually increased. In facilities that had not run classes prior, the SUp classes were additional as the extra staff time was paid for and the physical therapist’s usual case load was not affected. When funding was stopped status quo returned to the system (LTC), with physical therapist applying some elements of SUp to their work but not delivering SUp as designed. The sustainability of falls prevention programs beyond external funding is an ongoing problem [45]. Partnerships and collaborations, supported by policy have been identified as critical elements for sustainability [46]. What is not clear in the literature is why there appears to be an expectation that falls prevention can be delivered without appropriate ongoing financial resourcing. With the large cost of falls to the health system being known, surely it is cheaper to prevent a fall than pay to deal with the consequences.

### Strengths and limitations

A strength of this study was the sample of staff through the levels of LTC organizations from senior managers in corporate offices to clinicians providing care for residents in facilities. This diversity of roles represented in the sample enabled experiences of SUp to be explored from different perspectives and triangulated to gain a fuller understanding of what might help or hinder embedding SUp in everyday practice in LTC. These findings will contribute to program implementation decision making should SUp prove to be an effective falls prevention exercise program. While it was planned to include residents and their families, the sample did not include residents as the NZ COVID-19 restrictions impacted this study. During this time visitors were not being permitted to LTC facilities and therefore researchers were not able to run resident focus groups or gain resident permission to contact their family. Lockdown protocols also increased staff workload and some participants chose to be interviewed together, saving time, and resulting in unplanned focus groups. Power relationships from the staff hierarchy (nurse manager, nurse) may have played out with participants not speaking as freely as they may otherwise have in an interview.

## Conclusions

The results of this study suggest that if SUp is effective in preventing falls in LTC it may not become embedded within everyday practice, as designed, without additional funding support. This study identifies that policies that underpin funding decisions need to support physical therapy-led falls prevention exercise programs to be embedded in LTC. To be funded and resourced appropriately, the LTC facility service specifications need to be updated to recognise the health issue of falls in this population and current best practice evidence in LTC falls prevention. However, if status quo remains and funding is not attainable, the essential components of SUp need to be identified and a complexity informed approach taken to work with individual facilities to adapt SUp to suit.

## Supplementary Information

**Additional file 1.** Interview guides. The semi-structured interview guides that were used for interviews and focus groups.

## Abbreviations

FAC: Exercise group facilitator
HDEC: Health and Disability Ethics Committee
ID: Interpretive Description
interRAI: International Resident Assessment Instrument
LTC: Long-term care
Mgmt: Onsite management
NZ: New Zealand
RCT: Randomized controlled trial
RUG-III: Resource Utilisation Group
SRQR: Standards for Reporting Qualitative Research
SUp: Staying UpRight
SM: Senior management
