# Supplementary material for: “It’s all about the money”: an interpretive description of embedding physical therapy-led falls prevention group exercise in long-term care
Source: BMC Geriatr. 2023 Jan 11;23:14. doi: 10.1186/s12877-022-03722-z (PMC9832407; doi:10.1186/s12877-022-03722-z)
Supplement: Supplementary file 1 — Additional file 1. Interview guides. The semi-structured interview guides that were used for interviews and focus groups. [file 12877_2022_3722_MOESM1_ESM.docx]

**Additional file 1.** Interview guide for interviews and focus groups

Senior manager interview

How many years have you been in this role?

How many years have you been with the business?

o Can you tell me why you agreed to [name of organisation] taking part in the trial?

o Can you tell me what support was needed for the trial to run in [name of organisation]?

o How did staff feel about the trial/Was there any pushback from the staff about taking part? If yes how managed?

o Can you tell me what happened when the trial funding changed from external to within the organisation?

o Would you continue the Staying UpRight classes after the study?

o What are your views on making this programme sustainable within [name of organisation]? What changes would need to be made?

o Has there been any policy change as a result of this study?

o Can you tell me about any experiences/learnings from the COVID-19 pandemic that will be used in the future?

o Any other comments about the trial/intervention?

Onsite Management interview

How many years have you been in this role?

How many years have you been with the business?

o Can you tell me why you agreed to [name of of facility] taking part in the trial?

o How were you informed about the trial?

o Can you tell me how your facility got involved in the study?

o Can you tell me what support was needed for the trial to run in [name of of facility]?

o How did staff feel about the trial/Was there any pushback from the staff about taking part? If yes how managed?

o What are your views on falls prevention?

o What does your organisation value?

o Would you continue the Staying UpRight classes after the study?

o What are your views on making this programme sustainable within [name of of facility]? What changes would need to be made?

o What are your views on what Staying UpRight offers residents compared to what is routinely offered to residents?

o Did you share with other homes how the trial was working in your home?

o Can you tell me about any experiences/learnings from the COVID-19 pandemic that will be used in the future?

o Any other comments about the trial/intervention?

Exercise group facilitator Flex & Stretch Focus Groups

How many years have you been in this role?

How many years have you been with the business?

What is your professional background?

o What are your views on the training to deliver the class? (length of time? Manual? Ongoing support?)

o What else would you prefer?

o What are your views of the exercise classes in the trial? (content, duration, any different from usual classes?)

o How did the exercise groups affect workload, burden, and space (resource)?

o What were the factors that determined the length of the class?

o Can you tell me about what changes had to occur for the Staying UpRight group to run?

o Would you/What are your thoughts about continuing the Staying UpRight classes after the study?

o What changes did you see in the residents taking part over the course of the trial?

o Can you tell me what changes need to be made to the classes if they were to continue?

o Can you tell me about any experiences/learnings from the COVID-19 pandemic that will be used in the future?

o What went well and what were the barriers to the classes?

o What strategies did you use to address those barriers?

o Any other comments about the intervention?

Exercise group facilitator Staying UpRight Flex & Stretch Focus Groups

How many years have you been in this role?

How many years have you been with the business?

o What are your views on the training to deliver the class? (length of time? Manual? Ongoing support?)

o What are your views of the exercise classes in the trial? (content, duration, any different from usual classes?)

o How did the exercise groups affect workload, burden, and space (resource)?

o What did you think did/not contribute to the class running smoothly?

o What are your views on what Staying UpRight offers residents compared to what is routinely offered to residents?

o What changes did you see in the residents taking part over the course of the trial?

o Would you/What are your thoughts about continuing the Staying UpRight classes after the study?

o Can you tell me what changes need to be made to the classes if they were to continue?

o Can you tell me about any experiences/learnings from the COVID-19 pandemic that will be used in the future?

o Any other comments about the intervention?
